# Clinical outcomes of gemcitabine-loaded callispheres drug-eluting beads for patients with advanced and inoperable lung cancer: A case series study

**DOI:** 10.3389/fphar.2022.992526

**Published:** 2022-09-29

**Authors:** Yonghua Bi, Bo Zhang, Jianzhuang Ren, Xinwei Han, Wenze Wu

**Affiliations:** ^1^ Department of Interventional Radiology, The First Affiliated Hospital of Zhengzhou University, Zhengzhou, China; ^2^ Department of Interventional Radiology, Jingzhou Central Hospital, The Second Clinical Medical College of Yangtze University, Jingzhou, China

**Keywords:** lung cancer, drug-eluting beads transarterial chemoembolization (DEB-TACE), callispheres beads, gemcitabine, transarterial chemoembolization (TACE)

## Abstract

**Aim:** Drug-eluting beads transarterial chemoembolization (DEB-TACE) has not been widely used in patients with advanced and inoperable lung cancer. We aimed to report the preliminary outcomes of DEB-TACE with gemcitabine-loaded CalliSpheres beads for patients with advanced and inoperable lung cancer.

**Methods:** From November 2017 to October 2021, 37 patients (29 males, mean age 64.7 ± 10.3 years) with advanced and inoperable lung cancer underwent DEB-TACE with gemcitabine-loaded CalliSpheres beads. The primary endpoint was overall response rate, and the secondary endpoints were overall survival and progression-free survival.

**Results:** A total of 54 sessions of DEB-TACE were performed in 37 patients, with a technique success rate of 100%. Fourteen patients received a second session of DEB-TACE. The mean follow-up time was 18.7 ± 11.9 months. After 1, 3, and 6 months, overall response rate and disease control rate were 27.8% and 91.7%, 25.8% and 74.2%, 32.1%, and 67.9%, respectively. The median progression-free survival was 8.8 months (95% CI 7.5, 12.5 months). The 3-, 6- and 12- month progression-free survival rates were 67.1%, 57.0%, and 30.1%, respectively. The median overall survival was 10.0 months (95% CI 4.5, 13.1 months). The 3-, 6-, and 12- month overall survival rates were 88.5%, 72.7%, and 40.9%, respectively. Minor complications were observed in 14 patients (37.8%), with no procedure-related deaths or severe adverse events.

**Conclusion:** DEB-TACE with gemcitabine-loaded CalliSpheres beads is a safe, feasible and effective treatment strategy for patients with advanced and inoperable lung cancer.

## Introduction

Lung cancer is a common leading cause of deaths worldwide. Platinum-containing regimens (Schiller et al., 2002) or non-platinum-containing regimen (Pujol et al., 2005) are recommended in the treatment of advanced non-small-cell lung cancer (NSCLC). Transarterial chemoembolization (TACE) has been used for locally advanced non-small cell lung cancer after failure of concurrent chemoradiotherapy (Chen et al., 2020). As a novel palliative treatment, drug-eluting beads TACE (DEB-TACE) can not only embolize tumor supply vessels, but also slowly release the loaded chemotherapeutic drugs (Bi et al., 2021a). CalliSpheres bead is the first DEB product made in China. DEB-TACE with CalliSpheres beads has been widely used for advanced and inoperable hepatocellular carcinoma (Zhou et al., 2018; Xiang et al., 2019) and sometimes used for advanced or recurrent cervical cancer (Bi et al., 2021d) and so on (Bi et al., 2021b; a; Bi et al., 2022a; Bi et al., 2022b). It is reported that DEB-TACE using CalliSpheres beads is efficient and well tolerated in patients with hepatocellular carcinoma, which achieves better progression-free survival and treatment response while equal safety compared to conventional TACE (Zhou et al., 2018; Xiang et al., 2019).

DEB-TACE with pirarubicin-loaded CalliSpheres (Shang et al., 2020; Bi et al., 2021c; Lin et al., 2021) or cisplatin-loaded HepaSphere (Yu and Hu, 2020) has been used for advanced lung cancer. Currently, few studies reported the clinical outcomes of gemcitabine-loaded CalliSpheres beads for advanced and inoperable lung cancer. In a previous case report (Bie et al., 2019), DEB-TACE loaded with gemcitabine (800 mg) using CalliSpheres beads and transarterial infusion with nedaplatin (80–100 mg) was performed in only 6 patients. However, pirarubicin or nedaplatin are not standard regimens for advanced lung cancer (Schiller et al., 2002). In this current study, we performed DEB-TACE using gemcitabine-loaded CalliSpheres beads and transarterial infusion with cisplatin/carboplatin or docetaxel for the treatment of advanced and inoperable lung cancer, and we aimed to assess its safety and clinical efficacy.

## Patients and methods

### Study design

Ethical approval was waived by the Institutional Review Board of University due to its retrospective characteristics. Written informed consent was obtained from all patients. This current retrospective study enrolled 37 patients with advanced and inoperable lung cancer who received DEB-TACE with gemcitabine-loaded CalliSpheres beads from November 2017 to October 2021. Indications for DEB-TACE: age range from 18 to 85 years; pathological diagnosis of lung cancer, local progression after standard treatments; ineligible or refused to receive standard treatments due to emphysema, heart disease, or severe pulmonary fibrosis; without other life-threatening diseases. Exclusion criteria: combined with other malignant tumor but with no curative treatment; lower white blood cell count (< 3.0 × 10^9^/L) or lower platelets (< 40.0 × 10^9^/L); severe infection; pregnant or breastfeeding women. DEB-TACE treatment for lung cancer patients is discussed and individually tailored. The final choice of DEB-TACE strategy needs to consider the factors such as the treatment willingness and economic ability of the patients and their families.

### Data collection

Patients’ data were retrospectively collected and analyzed, such as clinical/demographic features, Eastern Cooperative Oncology Group (ECOG) performance status, the tumor-node-metastasis (TNM) classification, illness history, location and size of tumor, DEB-TACE procedure, tumor markers, adverse events, data of computed tomography, and so on (Figures 2A–C; Figures 3A,B; Figures 4A,B). The Global Initiative for Chronic Obstructive Lung Disease (GOLD) defines a standard for pulmonary severity levels based on the predicted ratio of forced expiratory volume in one second (FEV1%) values (Rabe et al., 2007).

### CalliSpheres beads

**FIGURE 1:**
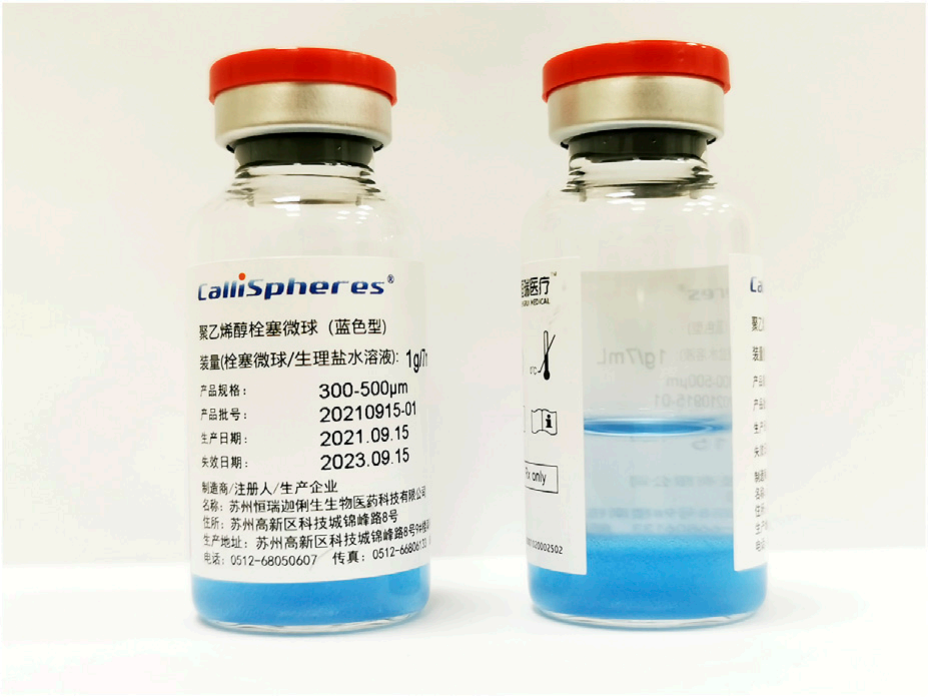
Photograph of CalliSpheres beads. It is a blue polyvinyl alcohol particles beads with a smooth surface, uniform size, and usually100–300 μm or 300–500 μm in diameter. About 7 ml of physiological sodium chloride solution and 2 ml beads were kept in bottles with no pharmacological agent.

A CalliSpheres bead (Jiangsu Hengrui Medicine Co. Ltd. Jiangsu, China) is a type of ion-exchange bead with some negatively charged functional groups, which are responsible for the loading of some positively charged drugs (Zhang et al., 2017). It is a blue polyvinyl alcohol particles beads with a smooth surface, uniform size, and usually 100–300 μm or 300–500 μm in diameter. About 7 ml of physiological sodium chloride solution and 2 ml beads were kept in bottles with no pharmacological agent (Figure 1). The pharmacological agent are loaded extemporaneously about 30 min before DEB-TACE.

### DEB-TACE procedures

**FIGURE 2:**
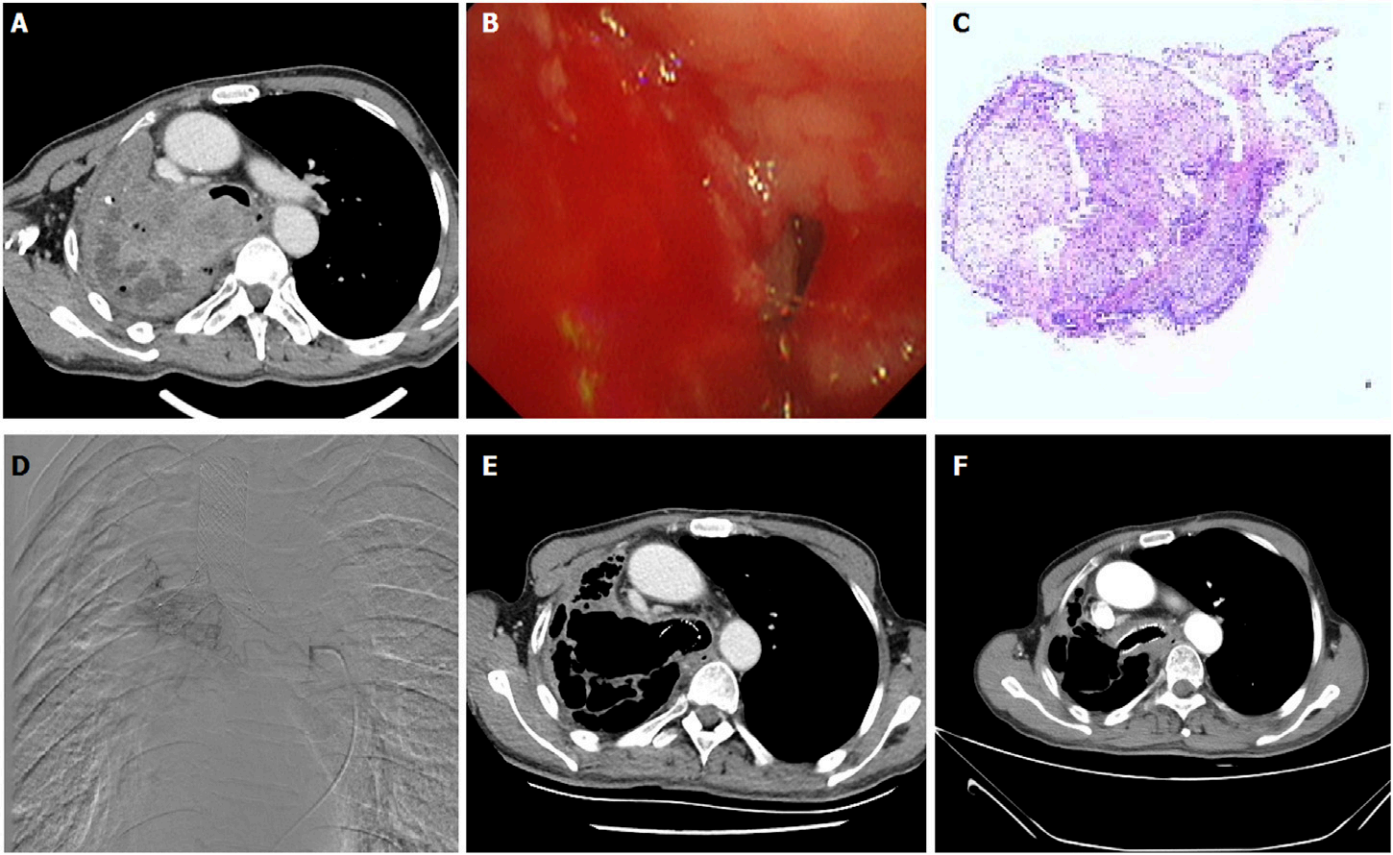
A 65-years male treated by CalliSpheres beads for advanced squamous cell carcinoma. **(A)** Computed tomography examination on admission revealed malignant tumor, severe bronchial stenosis and complete atelectasis of right lung. **(B)** Fibrobronchoscopy revealed a right main bronchial occlusion. **(C)** Pathological diagnosis of squamous cell carcinoma by biopsy of the lesion. **(D)** The right bronchial artery was the blood supply artery of the tumor, and was embolized by drug-loaded beads. **(E)** The right lung tumor was found to shrink after 1.5 months’ follow-up. **(F)** The airway stent was in good position and the tumor was almost disappeared after 1 year.

**FIGURE 3:**
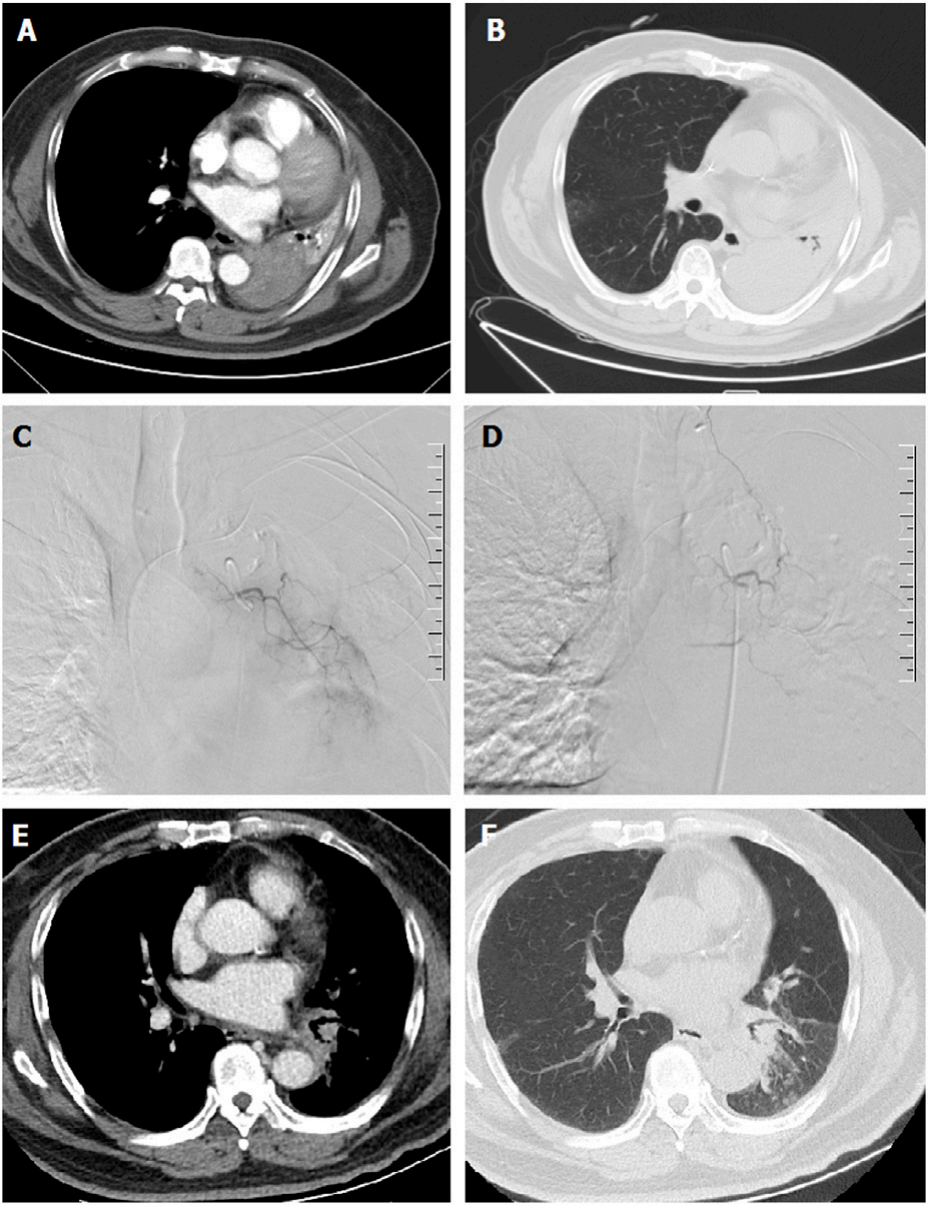
A 67-years male treated by CalliSpheres beads for advanced squamous cell carcinoma. **(A,B)** Computed tomography preoperative examination revealed a large tumor of the left lung with complete atelectasis. **(C,D)** The left bronchial artery was super-selectively incubated and embolized by drug-loaded beads. **(E,F)** The left lung tumor was found to shrink and the left lung atelectasis disappeared after 2 weeks’ follow-up.

**FIGURE 4:**
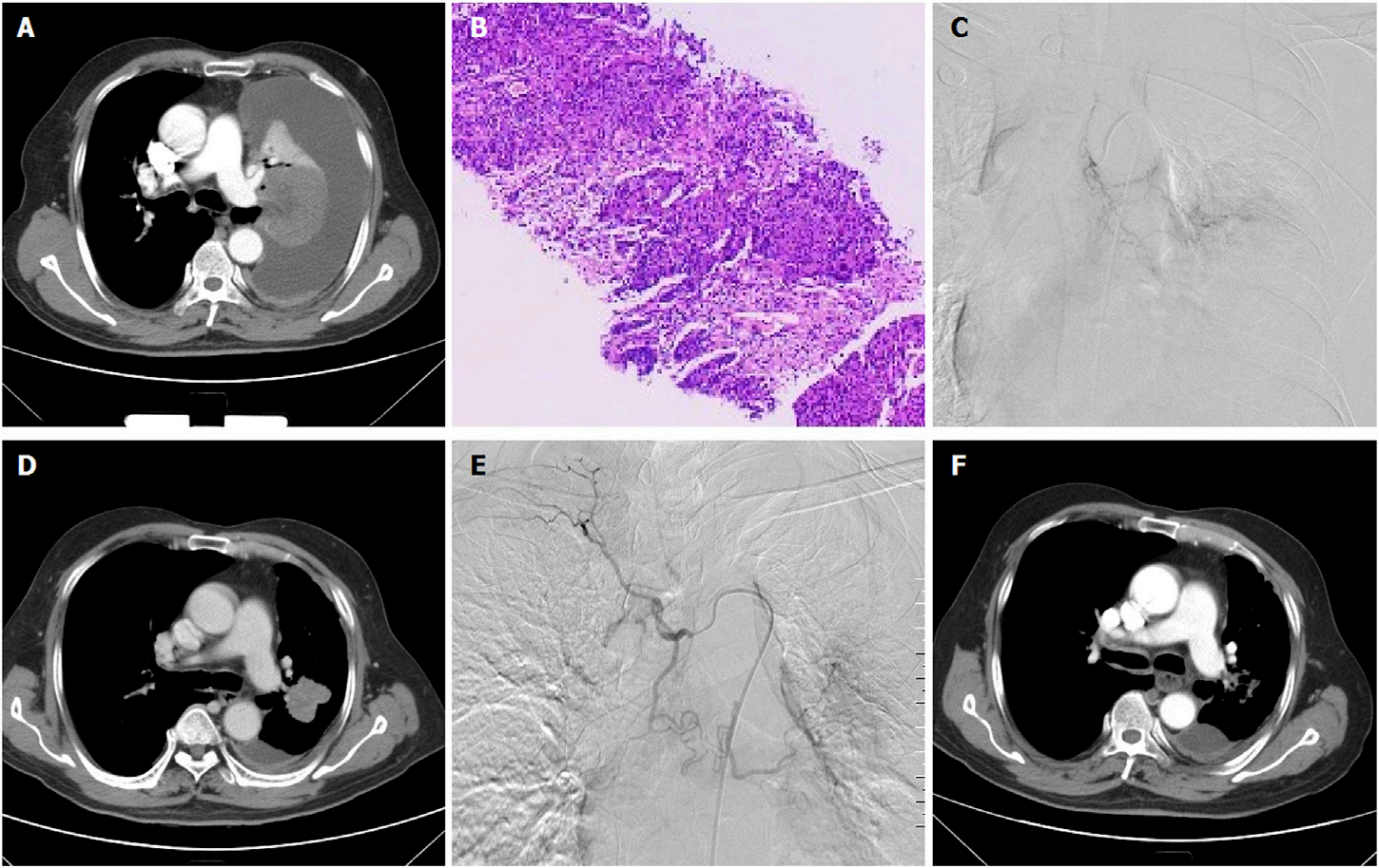
A 66-years male treated by CalliSpheres beads for advanced squamous cell carcinoma in left lung. **(A)** Computed tomography examination before procedure revealed malignant central tumor of left lung, with complete atelectasis and massive pleural effusion. **(B)** Pathological diagnosis of squamous cell carcinoma by biopsy of the lesion. **(C)** The left lung tumor was treated by CalliSpheres beads. **(D)** The left lung tumor shrunk and atelectasis disappeared 1 month after the first session of DEB-TACE. **(E)** The left tumor was treated again by drug-loaded beads 1 month later. **(F)** The tumor was almost disappeared 3 months after first DEB-TACE.

Under local anesthesia, the right femoral artery was accessed and a 5F-Cobra catheter (Terumo, Japan) was introduced. Tumor-feeding vessels were catheterized and embolized, including bronchial arteries, internal thoracic arteries, phrenic arteries. A 2.7-F microcatheter (Progreat, Terumo, Japan) was used for super-selection of all tumor-feeding arteries. Cisplatin or carboplatin was initially infused for patients with no previous platinum-based chemotherapy. Docetaxel was infused for patients receiving non-platinum-containing regimen. Gemcitabine (0.4–1.0 g) was pre-loaded with CalliSpheres beads for 30 min, and then mixed with iodixanol (ratio 1:1) as contrast developer before embolization. CalliSpheres beads were slowly injected into the tumor-feeding vessels under fluoroscopic guidance. One vial of CalliSpheres beads embolization was used for each patient, additional embolization was performed by using polyvinyl alcohol particles (Merit, American) or gelatin sponge particles if embolization is insufficient (Figure 2D; Figures 3C,D; Figures 4C,E).

### Endpoint

The primary endpoint was overall response rate, which was assessed by chest computed tomography before procedure and during follow up. The overall response rate was defined as the sum of complete response and partial response. Disease control rate was calculated as the sum of complete response, partial response and stable disease. The secondary endpoints were overall survival and progression-free survival.

### Safety assessment

According to the Common Terminology Criteria for Adverse Events (CTCAE) (version 4.0) (National Cancer Institute NIH, US Department of Health and Human Services, 2009), serious adverse events and adverse events were recorded perioperatively and during the follow up.

### Follow-up

Patients were followed up by chest computed tomography about 1, 3 and 6 months after DEB-TACE (Figures 2E,F; Figures 3E,F; Figures 4D,F). Patients were followed up by phone calls, and the last follow-up date was 17 November 2021.

## Results

### Patient characteristics

A total of 37 patients were enrolled in this study, including 29 males and 8 females (mean age 64.7 ± 10.3 years, range 38–83 years). As shown in Table 1, 27 patients (73.0%) were diagnosed with squamous cell carcinoma and 6 patients (16.2%) were adenocarcinoma. Local or distant metastases were present in 25 patients (67.6%). Atelectasis and/or obstructive pneumonia were found in 17 patients (45.9%). Five patients showed symptomatic relief in atelectasis and/or obstructive pneumonia about 1–2 months after DEB-TACE. Twenty-eight patients complained of cough or expectoration, and median duration of symptom was 6.0 months. Sixteen patients showed symptomatic relief in cough or expectoration about 1–2 months after DEB-TACE. A total of 16 patients (43.2%) received other synchronous treatments, such as radiotherapy and/or chemotherapy (*n* = 15), targeted therapy (*n* = 3) or immunotherapy (*n* = 5). There were 13 GOLD one patients, and the remained 24 patients showed normal pulmonary function.

**TABLE 1:** Patient characteristics at admission.

Variables	Data
Male, *n* (%)	29 (78.4%)
Mean age, years	64.7 ± 10.3
Lesion types	
Squamous cell carcinoma	27 (73.0%)
Sarcoma	1 (2.7%)
Adenocarcinoma	6 (16.2%)
Others	3 (8.1%)
TNM staging	
ⅡA/ⅡB	0 (0.0%)/4 (10.8%)
ⅢA/ⅢB/ⅢC	1 (2.7%)/10 (27.0%)/2 (5.4%)
ⅣA/ⅣB	10 (27.0%)/10 (27.0%)
Median duration of symptom	6.0 (1.0, 11.0)
ECOG score: 0/1/2	16 (43.2%)/18 (48.6%)/3 (8.1%)
GOLD stage 1	13 (35.1%)
Radiotherapy and/or chemotherapy	15 (40.5%)
Targeted therapy/immunotherapy	3 (8.1%)/5 (13.5%)
Atelectasis and/or obstructive pneumonia	17 (45.9%)
Hemoptysis	11 (29.7%)
Single tumor/multiple tumors	26 (70.3%)/11 (29.7%)
Central/peripheral location	18 (48.6%)/19 (51.4%)
Local or distant metastasis	25 (67.6%)
Tumor diameter, mm	57.0 ± 33.5
Pre-operative laboratory tests	
WBC, normal 4–10 × 10^9^/L	7.9 (6.0, 9.4)
AFP, normal 0–10 ng/ml	2.1 (1.5, 2.9)
CEA, normal 0–4 ng/ml	4.5 (2.4, 8.2)
Cyfra 21–1, normal 0–3.3 ng/ml	4.1 (1.9, 8.7)
CA153, normal 0–30 U/mL	18.0 (4.4, 25.1)
CA125, normal 0–35 U/mL	23.3 (17.3, 61.6)
CA19-9, normal 0–37 U/mL	9.0 (4.3, 19.5)

TNM, tumor-node-metastasis; ECOG, Eastern Cooperative Oncology Group; GOLD, Chronic Obstructive Lung Disease; WBC, White blood cell; AFP, Alpha fetoprotein; Cyfra, cytokeratins, non-small cell lung cancer antigen; CA, Carbohydrate antigen.

### DEB-TACE treatments

A total of 54 sessions of DEB-TACE were performed in 37 patients, and 14 patients received a second session of DEB-TACE, with a mean of 1.5 ± 0.7 sessions per patient. All DEB-TACE procedures were successfully performed, with a technique success rate of 100%. Fourteen Patients were all treated with gemcitabine-loaded DEB-TACE. The median dose of gemcitabine was 0.5 ± 0.2 g. A total of 98 tumor supply vessels were embolized, including 62 bronchial arteries (double bronchial arteries in 11 sessions), 27 internal thoracic arteries, three phrenic arteries and 6 other arteries. Except for one session of DEB-TACE with CalliSpheres beads of 100–300 μm, the remaining sessions of DEB-TACE was performed by using CalliSpheres beads of 300–500 μm in diameter. All patients received a bottle of beads, 16 of whom (43.2%) received additional embolization. Polyvinyl alcohol or gelatin sponge particles of 350–560 μm were used in 10 and 5 patients after DEB-TACE. Thirteen patients (35.1%) received other treatments, of which, 6 patients underwent airway stent placement due to malignant airway stricture. Eight patients underwent ^125^I seeds implantation and two patients underwent upper vena cava stenting. The median inpatient duration was 13.0 days and the mean cost of hospitalizations was 6.8 × 10^4^ Renminbi (Table 2).

**TABLE 2:** Clinical data on DEB-TACE.

Variables	Data
Median dose of gemcitabine, g	0.5 ± 0.2
Cisplatin, mg	55.5 ± 10.4
Carboplatin, mg	243.3 ± 98.3
Docetaxel, mg	44.0 ± 8.9
Polyvinyl alcohol particles	10 (27.0%)
Gelatin sponge particles	5 (13.5%)
Embolization microspheres	3 (8.1%)
Median inpatient duration, months	13.0 (8.8, 16.0)
Mean cost of hospitalization, × 10^4^ Renminbi	6.8 ± 2.8
Mean session of DEB-TACE	1.5 ± 0.7
Mean procedure time, min	88.6 ± 33.5
Complications, *n* (%)	14 (37.8%)
Fever	4 (10.8%)
Nausea and/or vomiting	5 (13.5%)
Chest pain	7 (18.9%)
Chest stuffiness	2 (5.4%)
Angina pectoris attacks	1 (2.7%)
Other treatments, *n* (%)	
Airway stenting	3 (8.1%)
Upper vena cava stenting	2 (5.4%)
^125^I seeds implantation	8 (21.6%)
Inferior vena cava filter	1 (2.7%)

### Endpoint

**FIGURE 5:**
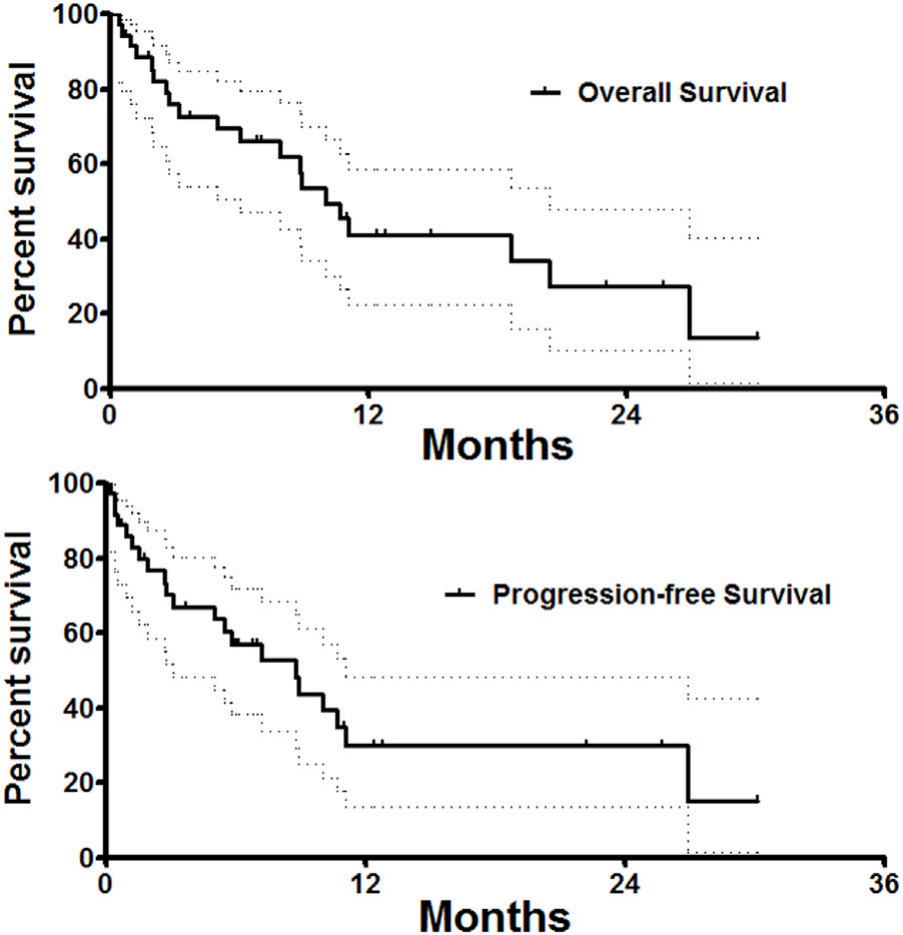
Follow up. Median overall survival and progression-free survival was 10.0 and 8.8 months. The 3-, 6 and 12 months overall survival rates were 88.5%, 72.7%, and 40.9%, respectively.

Only one patient was lost to follow-up, the median follow-up time was 18.7 ± 11.9 months. As shown in Table 3, overall response rate and disease control rate were 27.8%, 91.7%, 25.8%, 74.2%, 32.1%, and 67.9% respectively at 1, 3, and 6 months after DEB-TACE. The median progression-free survival was 8.8 months (95%CI 7.5, 12.5 months). The 3-, 6- and 12 months progression-free survival rates were 67.1%, 57.0%, and 30.1%, respectively. The median overall survival was 10.0 months (95%CI 4.5, 13.1 months). The 3-, 6 and 12 months overall survival rates were 88.5%, 72.7%, and 40.9%, respectively (Figure 5).

**TABLE 3:** Local tumor response.

Response	1 month	3 months	6 months
Complete response	1 (2.8%)	3 (9.7%)	3 (10.7%)
Partial response	9 (25.0%)	5 (16.1%)	6 (21.4%)
Stable disease	23 (63.9%)	15 (48.4%)	10 (35.7%)
Progressive disease	3 (8.3%)	8 (25.8%)	10 (35.7%)
Overall response rate	10 (27.8%)	8 (25.8%)	9 (32.1%)
Disease control rate	33 (91.7%)	23 (74.2%)	19 (67.9%)

### Safety

Minor complications were observed in 14 patients (37.8%), with no severe adverse events, such as procedure-related deaths, nontarget embolization, catheter-related adverse events, or massive bleeding. Minor complications were observed in 14 patients (37.8%). Only one patient (2.7%) had angina pectoris attacks about half an hour after the second session of DEB-TACE procedure. This patient had a history of hypertension for many years, a history of coronary heart disease bypass surgery, and had acute myocardial infarction more than 1 month ago. It showed decreased oxygen saturation, increased heart rate and increased blood pressure, and symptoms relieved the symptoms after antihypertensive, diuretic and dexamethasone. Five patients showed nausea and/or vomiting and seven patients (18.9%) had mild chest pain, those patients were well controlled within 2–3 days.

## Discussion

Platinum-containing regimens (cisplatin + paclitaxel, cisplatin + gemcitabine, cisplatin + docetaxel, or carboplatin + paclitaxel) (Schiller et al., 2002) or non-platinum-containing regimen (gemcitabine–docetaxel combination) (Pujol et al., 2005) are recommended in the treatment of advanced NSCLC. According to the National Comprehensive Cancer Network (NCCN) Guidelines Insights (Ettinger et al., 2021), several new targeted therapies or new indications for therapies, including capmatinib, lorlatinib, pralsetinib, selpercatinib, and fam-trastuzumab deruxtecan, are now recommended for eligible patients with metastatic NSCLC who have certain actionable biomarkers. Lorlatinib is recommended as another preferred first-line therapy option for patients with ALK rearrangement–positive metastatic NSCLC. Entrectinib is recommended as a subsequent therapy option for patients with ROS1-positive disease who have central nervous system progression after crizotinib.

As a novel delivery and embolization system, DEB-TACE can not only embolize tumor-feeding vessels to block the blood supply of lung cancer, but also increase the retention time and drug concentration of antitumor drugs by mean of slow release. Thus, DEB-TACE can theoretically both increase effectiveness of tumor necrosis and reduce systemic drug concentration and adverse events (Kunisada et al., 2013; Choi et al., 2014; Mikhail et al., 2018). Yu and Hu (2020) performed DEB-TACE with cisplatin-loaded HepaSphere and concurrent transarterial infusion with docetaxel (120 mg) for a 69-year-old male with locally advanced squamous non-small cell lung cancer. DEB-TACE with pirarubicin-loaded CalliSpheres beads have been used for advanced lung cancer (Shang et al., 2020; Bi et al., 2021c; Lin et al., 2021); however, pirarubicin is not standard regimens (Schiller et al., 2002). Nowadays, few studies reported the clinical outcomes of gemcitabine-loaded CalliSpheres beads for advanced and inoperable lung cancer. In a previous case report of Bie et al. (2019), DEB-TACE was performed with gemcitabine (800 mg) loading CalliSpheres beads and transarterial infusion with nedaplatin (80–100 mg). However, only 6 patients received DEB-TACE and nedaplatin is not standard regimens for advanced and inoperable lung cancer (Schiller et al., 2002).

In this current study, we performed DEB-TACE using gemcitabine-loaded CalliSpheres beads and transarterial infusion with cisplatin/carboplatin or docetaxel for the treatment of advanced and inoperable lung cancer. After 1, 3, and 6 months, overall response rate and disease control rate were 27.8%, 91.7%, 25.8%, 74.2%, 32.1%, and 67.9%, respectively. The median progression-free survival and the 6-months progression-free survival rate after DEB-TACE were 8.8 and 57.0%, respectively. Our data was similar to that of DEB-TACE with gemcitabine-loaded CalliSpheres beads in a previous case report (Bie et al., 2019). The median progression-free survival was 8.0 months and the 6-months progression-free survival rate was 66.7%. Our preliminary data indicated that DEB-TACE with gemcitabine-loaded CalliSpheres beads was associated with good disease control during a short-term follow-up period.

A larger tumor volume and failure previous systematic treatments may predict a poorer therapeutic response to DEB-TACE, and a combination of other therapies can improve the disease prognosis (Bie et al., 2019). For example, a combined radioactive iodine-125 implantation was associated with a better prognosis (Yang et al., 2016; Chen et al., 2017). In this study, 8 patients received ^125^I seeds implantation after progressive disease. Besides, it is also important to treat severe complications caused by tumor progression, such as severe airway stenosis (Bi et al., 2020). In this study, three patients underwent airway stenting and two patients underwent upper vena cava stenting.

Regarding the safety, no serious adverse events or procedure-related death were observed in this study. Minor complications were observed in 14 patients (37.8%). Only one patient (2.7%) had angina pectoris attacks after procedure. The symptoms relieved after antihypertensive, diuretic and dexamethasone. DEB-TACE using gemcitabine loaded CalliSpheres beads appear to be safe for patients with advanced and inoperable lung cancer.

There are some limitations. This is a retrospective observational study performed in a single center. There is more than one medication regimen, which is not conducive to drawing a clear conclusion. The limited sample size does not allow for statistically powered subgroup analysis. More studies are wanted to verify the safety and efficacy. In conclusion, DEB-TACE with gemcitabine-loaded CalliSpheres beads is safe, feasible and well-tolerated for patients with advanced and inoperable lung cancer.

## Data Availability

The original contributions presented in the study are included in the article/Supplementary Material, further inquiries can be directed to the corresponding authors.
